# Religious Psychology—St. Anthony of Padua

**Published:** 1900-11

**Authors:** 


### RELIGIOUS PSYCHOLOGY—ST. ANTHONY OF PADUA.

(Translated by Bernard Wolff, M.D., from *L'Indépendance Médicale.*)

Anthony was born at Lisbon in the year 1195, of parents noted for the nobility of their birth and for their piety: Martin de Bouillon, descendant of Godfrey de Bouillon, who was made king of Jerusalem, and Maria Theresa de Travera of Troila. He was given the name of Fernandez, which he changed to Anthony upon his entrance into holy orders. He first received instruction from the canons of the cathedral of Lisbon.

At the age of fifteen he lost his parents. He then entered the monastery of Saint Vincent, near Lisbon, occupied by the friars of St. Augustine. But he was continually annoyed by the visits and objurgations of his family, so that he obtained permission to go to St. Croix of Coimbra, where he delivered himself up with ardor to his oraisons. At this time occurred his first miracles, or, better, his first hallucinations. They are described by his companion, Brother Luke: "I was myself," he says, "witness to a great number of these miracles, which, unless one saw them, one could scarcely believe could have been performed by mortal man, even in a sanctified state. But the multitude of miracles which I shall record may not be doubted, for I bear good and faithful witness, for I have never lied in anything. And this I do not say for self-praise, but that my word may not be placed in doubt when I come to witness the miracles of our noble saint, and these miracles are such as there were never more marvelous, except those of our seraphic father Saint Francis."

Though he strove to separate himself from the things of earth that he might know himself, and that we might see him from a distance, Brother Luke lacked neither good sense nor perspicacity. Despite his admiration and respect for St. Francis, he did not fear to criticize his choice of Brother Eli as a companion. Brother Eli was, indeed, a person little to be commended, who played to the death of the saint a very equivocal *rôle*. It is, then, the narrative of Brother Luke which we shall follow for the most part in analyzing the life and character of the monk of Padua.

The first miracle is plainly only a hallucination, easily explainable from the mental state of a young person much exalted by prayer.

Anthony, detained one day by the performance of some menial task, could not attend the church where mass was being celebrated. Suddenly it seemed to him that the walls of the chapel split in twain to permit him to see the holy saints and to worship them. If his body was occupied with the work of the monastery, in spirit he was at the church, and, hence, there was nothing miraculous about the hallucination of which he was the victim.

The same explanation may be applied to the following vision reported by Father Luke: Anthony had heard of "poverello d'Ombrie" of "the holy Father Francis," and he ardently desired to enter among the minor brothers, as theirs was the most perfect school of poverty, humility and martyrdom. He had seen brought back to Coimbra, with great pomp, the bodies of five minor brothers who had been martyred in Morocco. In this state of mind it is not surprising to see visions produced in Anthony in conformity with these ideas, visions he very sincerely mistook for realities. One day he saw the soul of a minor brother from a neighboring monastery mount straight to heaven in a splendor of light. Again, he beheld before him the seraphic Father St. Francis, who was far away in Italy, and who said to him that he wished to make him a minor brother, and disclosed to him in what manner he was to serve the Holy Church in that venerable institution, and what a great number of souls he was to wrest from the claws of the devil. Naturally, Anthony interpreted these visions as counsels and encouragements. So he did not hesitate longer to enter the monastery of Olivarez.

After an unfortunate journey to Morocco, where he fell seriously ill, he heard that St. Francis was to hold a chapter general at Assisi. He obtained permission to go there and to receive the benediction of the founder of the order, which greatly increased his exaltation. Then began what might be called his public life and preaching.

At this time Brother Anthony was about twenty-six years old. If Brother Luke is to be believed he was of high stature, with a lofty forehead and large eyes, very soft and luminous. In reality it is very difficult to know exactly what was the appearance of St. Anthony, for in the different high reliefs of marble which decorate the Santo of Padua where his remains repose, his figure is variously depicted. In the first scene, by Antonio Minello, to the left at the side of the altar, he is represented as taking the vestments of a saint. He is nude, with the body and face of a child. In the second scene by Girolomo Campagna, the saint is beardless, with a figure fat and juvenile, almost that of a woman. He is represented in very much the same way in the scene sculptured by Jullio Lombardo, that of the stone being found in the place of a heart in the body of a miser. In the fifth scene by Minello and Sansovino, which shows the miraculous raising from the dead of a child, the face is nearly the same as in the other groups, except that he wears a small mustache and a slight beard on the chin. The face is different in the scene by T. Lombardo, the seventh, that of the healing of the broken leg, and also in the ninth scene from the same chisel, where the saint bears witness for a new-born child in favor of its mother. Here the face is thin and bony, with prominent nose and well-marked features. It is however generally believed that the portrait most resembling him is the bronze, by Donatello, which ornaments the principal altar of the Santo.

Anthony strove to conform his conduct to that of St. Francis of Assisi, and to imitate him in all things. To this end he wished to remain in Italy and to prepare himself for preaching by a diligent study of theology. He began peripatetic preaching, attracting multitudes, reclaiming drunkards and brigands, converting heretics and performing miracles on his way. The churches could not contain the crowds that pressed to hear him.

At Verceil during one of his sermons, the body of a young man was brought in the church, having not yet been buried. As his parents and friends wept and lamented over the premature death of the young man, Anthony began to pray and addressed himself to the dead man, saying, "In the name of the Lord, I command you to rise up and live." And immediately the young man arose. Brother Luke adds: "His cheeks were neither blanched nor sunken, as you would believe, but they were red and glowing with health," which is perhaps the explanation of the miracle.

Coming to France, there to preach against the Albigenses, he acquired the name of "Scourge of the Heretics." There also he performed many miraculous deeds. Brother Luke, over whom Anthony had obtained a great ascendency, saw him once singing in the choir of the monastery, when he was at the selfsame moment preaching in the cathedral pulpit, as he saw him later walking on the water of the canal of Venice, where, however, Luke prevented himself from following. "Brother Anthony," said he, "made the sign of the cross on the canal, and then began to walk on the water with as much assurance as though walking on a solid bridge, and then commanded me to follow him, promising me that no harm should come to me, but I did not wish to obey him, because the water was very deep and I have always held it in great aversion, and I preferred to pass over by the bridge, and if this is a sin against holy obedience I pray God to take away the fault."

The good Brother Luke lent himself readily to suggestion, and Anthony had over him a very considerable influence. He recounts that one day, being surrounded by heretics, in a spirit of mockery, a roasted toad was presented him to eat. "Anthony crossed himself," says Luke, "and having carved the horrible thing, presented me with a quarter of it, who protested that not even the power of holy obedience could induce me to swallow the detestable meat. But suddenly there appeared before me, instead of the horrible toad, the quarter of a fine pullet, so fat and beautifully browned by the fire that water came to my mouth on merely regarding it, and I was in haste to eat it. You may believe me that a more delicious morsel I never ate."

Nevertheless, the credulity of Brother Luke has its limits. When his biological interests were menaced he resisted, preferring, without hesitation, to sin against the holy obedience, as we have seen in the miracle at Venice.

Another instance of the same nature: The heretics requested Anthony and Luke one day to dine with them in their house, thinking to poison the holy brothers. Anthony discovered the plot through the agency of a vision from on high, and, while reproaching the heretics for their criminal design, he began to eat, telling Brother Luke to do likewise, assuring him that no evil would befall him. "But," said the latter, "I could not believe, not finding myself in that state of grace where I could hope that our Lord would be justified in performing a miracle to save my life. I preferred, rather, to endure my hunger, which was great, than to place myself in peril of a terrible death by eating the meat in which I knew a subtle poison had been secretly put."

---

### SOME EXPERIENCES WITH THE SCHLEICH MIXTURE.

P. Ilyin (*Klin. therap. Woch.*, July 29, 1900) has employed the Schleich solutions in one hundred and thirty-five cases, and is well satisfied with the results. All disagreeable symptoms of either narcosis, such as salivation and bronchitis, were but rarely noticed, and as long as respiration was active sudden death, which is liable to occur after chloroform administration, seemed to be impossible. Depending upon the age, from 0.98 to 2.70 cubic centimeters of the mixture were found necessary per minute to keep the patients under. Excitation was noticed in twenty-five cases, nausea in nineteen, convulsive movements in three, trismus in two, and temporary cessation of respiration in two, one of which was an extremely weak child suffering from post-scarlatinal osteomyelitis. Respiration becomes more frequent and deep, and is most rapid when complete narcosis has set in unless dyspnea is present, when an increased rate is not observed. There seems to be a close association between the degree to which the patient is anesthetized and the number of respirations, for when the patient is allowed to come out the normal rate gradually returns. The pulse is full and not rapid; as consciousness returns, it becomes more frequent and weak. The author believes that, owing to the physical properties of the mixture, one can regulate the amount within perfectly safe limits by carefully and constantly watching the respiration and circulation.—*Med. News.*

